# The Pathophysiology of Hereditary Angioedema

**DOI:** 10.1097/1939-4551-3-S3-S25

**Published:** 2010-09-15

**Authors:** Bruce L Zuraw

**Affiliations:** 1 Department of Medicine, University of California San Diego and San Diego Veteran's Affairs Medical Center, La Jolla, Cal

**Keywords:** HAE, C1 inhibitor, contact system, bradykinin, plasma kallikrein, Hageman factor

## Abstract

Hereditary angioedema (HAE) causes recurrent episodes of angioedema that may be very severe and are frequently associated with significant morbidity and even mortality. Understanding the pathophysiology of this disease is crucial for proper diagnosis and management of these patients. HAE is caused by mutations in the *SERPING1 *gene that result in decreased plasma levels of functional C1 inhibitor. A large number of different mutations have been described that result in HAE. About 15% of patients have a mutation at or near the active site of the reactive mobile loop, resulting in a protein that lacks functional activity (type II HAE). Type I HAE is caused by a diverse range of mutations, some of which cause the nascent protein to misfold and thus to be unable to enter the secretory pathway. The primary mediator of swelling in HAE is bradykinin, a product of the plasma contact system. Bradykinin induces increased vascular permeability by activating the bradykinin B2 receptor, which results in phosphorylation of vascular endothelial cadherin. The regulation of both the bradykinin B2 receptor and peptidases that degrade bradykinin may influence HAE disease severity. HAE results from mutations in the *SERPING1 *gene that lead to a loss of functional C1 inhibitor. Attacks of angioedema result from generation of bradykinin, which acts on bradykinin B2 receptors to enhance vascular permeability.

## 

In 1888, William Osler described a familial form of angioedema associated with significant morbidity and mortality[1]. He called this disease hereditary angio-neurotic edema (HANE), a name that has subsequently been shortened to hereditary angioedema (HAE). The prevalence of HAE is not known for certain, but has been estimated to range from 1:30,000 to 1:80,000 in the general population without any known sex, ethnic, or racial differences[2].

HAE is clinically characterized by recurrent episodes of angioedema that typically involve the extremities, gastroin testinal tract, face, oropharynx, larynx, or external genitalia. Patients with HAE experience discrete episodes of nonpruritic nonpitting angioedema at these sites[2,3]. HAE attacks are classically distinguished from allergic or idiopathic angioedema by their longer duration (typically 72-96 hours), absence of accompanying urticaria and failure to respond to antihistamines or corticosteroid therapy.

Most often, patients with HAE become symptomatic during childhood. Fifty percent of HAE patients first experience a swelling episode before age 10, with some patients manifesting angioedema as early as 1 year of age[4,5]. Most patients then experience a worsening of symptoms around puberty[3]. Occasionally, patients with HAE do not begin to show evidence of angioedema until their late teens or early adulthood. Rare patients with HAE have been reported who never experience angioedema, but are identified through screening family members of a symptomatic patient[5,6]. Although some patients seem to experience decreased symptoms as they age, other patients continue to experience HAE attacks throughout their lives.

This article will address 4 questions that will help illuminate the clinical issues described above: 1) What is the underlying basis of HAE? 2) Why do patients become C1 inhibitor deficient? 3) What is the primary mediator of swelling in HAE? and 4) What is the molecular basis of bradykinin-induced swelling?

## What is the Underlying Basis of Hae?

The pathophysiologic basis of HAE as a deficiency of a plasma inhibitor was discovered in the early 1960s. Landerman et al reported that patients with HAE lacked an inhibitor of serum globulin permeability factor (shown subsequently to be activated Hageman factor) or plasma kallikrein[7]. The next year, Donaldson and Evans, in a seminal publication, identified the missing inhibitor as C1 inhibitor[8]. The identification of C1 inhibitor deficiency as the basis of HAE allowed the pathophysiologic and molecular mechanisms of HAE to be explored.

C1 inhibitor is a broad-spectrum serine protease inhibitor that is a member of the serpin (***ser***ine ***p***rotease ***in***hibitor) superfamily, with significant homology to a1-antitrypsin. It is the major inhibitor of several complement proteases (C1r, C1s, MASP-1, and MASP-2) and contact system proteases (plasma kallikrein and activated Hageman factor [coagulation factors XIIa and XIIf])[9]. C1 inhibitor is also an inhibitor of the fibrinolytic protease plasmin and the coagulation protease factor XIa.

Like other serpins, C1 inhibitor functions as a "molecular mousetrap," undergoing large scale rearrangement and trapping the target protease when it is cleaved[10]. C1 inhibitor is a suicide inhibitor, forming a 1:1 stoichiometric complex with the target protease, which is followed by clearance of the entire complex. In cases of overwhelming proteolytic activation, C1 inhibitor can be cleaved into a modified inactive form without forming a complex with or inhibiting the protease[11].

The 2.1-kb C1 inhibitor cDNA was initially cloned and sequenced in 1986[12]. C1 inhibitor is a 110-kD single-chain glycoprotein consisting of 478 amino acids plus a 22-residue signal peptide[12]. The protein is organized into 2 domains: the N-terminal 100 amino acids contain the N- and O-linked carbohydrates, and the C-terminal 378 amino acids contain the active site in a stressed loop conformation typical of the serpins[12]. The 17,159-basepair genomic sequence of the gene was published in 1991[13]. The gene, now named *SERPING1*, is located on chromosome 11 (p11.2-q13), contains 8 exons and 7 introns, does not contain a 5' TATA box, and is distinguished by 17 Alu repeats in introns 3 to 7. More recently, the crystal structure has been solved[14].

The C1 inhibitor deficiency in HAE has been shown to result from mutations of the *SERPING1 *gene. A large number of *SERPING1 *mutations have been identified,[9,15-18] with additional mutations still being reported. A database tabulating known *SERPING1 *gene mutations (http://hae.enzim.hu) currently lists more than 150 different mutations identified in patients with HAE.

The autosomal dominant pattern of HAE was recognized by Osler in 1888[1]. Each child of an affected patient has a 50% chance of having HAE. Importantly, HAE does not skip generations. Lack of a positive family history of angioedema cannot, however, be used to exclude the diagnosis. Although ~75% of patients give a history of having an affected parent, the remaining 25% of patients presumably have a de novo mutation of the C1 inhibitor gene that results in HAE[19].

## Why do Patients Become C1 Inhibitor Deficient?

Two different forms of HAE associated with low C1 inhibitor function have been identified[20]. Type I HAE is the most common form, accounting for about 85% of cases. It is associated with decreased antigenic levels of C1 inhibitor in the plasma. A large number of different *SERPING1 *mutations have been associated with type I HAE, including missense, nonsense, frameshift, deletion, and insertion mutations. In general, type I HAE is caused by mutations in the C1 inhibitor gene that result in either truncated proteins or misfolded proteins that cannot be secreted[21].

This failure of some type I C1 inhibitor mutant proteins to be efficiently secreted is presumably because of a failure of the mutant protein to correctly fold into the native C1 inhibitor structure. Newly synthesized proteins are cotranslationally translocated from the ribosome into the endoplasmic reticulum (ER), where they must fold into a native conformation before they are able to exit to the Golgi apparatus. Protein folding has been shown to follow a thermodynamic gradient in which the most stable or native conformation lies at a free energy minimum,[22] and specialized ER proteins (chaperones and folding enzymes) interact with the nascent protein, helping it to fold correctly and avoid kinetic traps until it reaches native conformation. If the protein fails to reach this conformation, it is no longer targeted for secretion; instead, it is retrotranslocated back into the cytosol for degradation in the proteasome[23].

Approximately another 15% of HAE patients have type II HAE. This type of HAE is characterized by normal plasma antigenic levels of C1 inhibitor but decreased functional levels of the plasma C1 inhibitor[20,24]. Most of *SERPING1 *mutations associated with type II HAE involve residues at or near the active site on the reactive mobile loop that result in a mutant C1 inhibitor protein that is secreted but is dysfunctional[25].

In both type I and type II HAE, the low functional level of C1 inhibitor results in diminished regulation of the complement and contact systems. Because C1 inhibitor functions as a suicide inactivator, every molecule of C1r, C1s, plasma kallikrein, or factor XIIa that is inhibited consumes a molecule of C1 inhibitor. Because of the reduced function of C1 inhibitor in HAE, turnover of the C1 inhibitor may be higher in patients with HAE[26]. Clearance of radiolabeled C1 inhibitor appears to support this hypothesis,[27] although pharmacokinetic analyses of infused C1 inhibitor concentrates have not confirmed the evidence of more rapid turnover of C1 inhibitor in HAE patients. This is an area that will require more information.

## What is the Primary Mediator of Swelling in Hae?

Identification of the mediator of swelling in patients with HAE has important implications for developing more effective treatments for the disease. Incubation of plasma from HAE patients ex vivo at 37°C generates a factor that causes smooth muscle contraction and increased vascular permeability[28]. It was quickly recognized that this vascular permeability enhancing factor was likely to be the mediator of swelling in HAE; however, the identity of this factor was an area of significant controversy for many years. C1 inhibitor is a major inhibitor of several complement proteases and contact system proteases. During HAE attacks, each of these plasma proteolytic cascades is activated and several vasoactive substances are potentially generated. From these considerations, 2 potential mediators of swelling in HAE were identified: C2 kinin, generated through activation of the classic complement pathway,[29] and bradykinin, generated through activation of the contact system[30].

Over time, compelling laboratory and clinical data have demonstrated that bradykinin is the primary mediator that enhances vascular permeability in HAE[11,30-39]. Bradykinin is a nanopeptide generated by activation of the contact system. In this system, active plasma kallikrein cleaves high-molecular-weight kininogen to release bradykinin. The generated bradykinin can potently increase vascular permeability by binding to its cognate receptor (the B2 bradykinin receptor) on vascular endothelial cells. Active plasma kallikrein has been detected in the blister fluid of HAE patients but not in that of normal controls[30]. Incubation of HAE plasma ex vivo was shown to generate bradykinin[31,32]. Furthermore, the contact system is activated in vivo during attacks of angioedema in HAE patients,[33-35] and increased levels of bradykinin have been measured in plasma during attacks of angioedema[36].

Contact system activation was also shown to cleave C1 inhibitor into a nonfunctional form,[11] and the plasma level of cleaved nonfunctional C1 inhibitor is increased during attacks of angioedema in HAE patients[37]. Importantly, the activity to enhance vascular permeability that is generated by ex vivo incubation of C1 inhibitor-deficient plasma was shown to be independent of C2 but completely dependent on high-molecular-weight kininogen (the substrate for bradykinin)[38]. C1 inhibitor knockout mice show a persistent increase in vascular permeability, which can be corrected by administration of exogenous C1 inhibitor[39]. Additionally, the vascular permeability defect depends on both plasma kallikrein activity and bradykinin receptor signaling[39]. In view of the overwhelming evidence supporting the predominant role of bradykinin as the mediator of swelling in HAE and the absence of substantiating evidence for a kinin derived from C2,[32,38] it was of little surprise that drugs targeting bradykinin generation or action have shown efficacy in clinical trials for treatment of acute attacks of angioedema in HAE[40,41].

## What is the Molecular Basis of Bradykinin-Induced Swelling?

With the demonstration that bradykinin is the mediator of swelling in HAE, more attention has been paid to the mechanisms that influence the ability of bradykinin to induce angioedema. In particular, the catabolism of bradykinin, the expression of bradykinin receptors, and the signaling events that result in swelling need to be considered.

Bradykinin is catabolized by a variety of peptidases (also called kininases). The most important peptidase in the catabolism of bradykinin is angiotensin converting enzyme (ACE); other important peptidases involved in the metabolism of bradykinin include aminopeptidase P (APP), carboxypeptidase N, neutral endopeptidase, and dipeptidyl peptidase IV[42,43]. Decreasing activity of these peptidases (such as by using an ACE inhibitor) can increase the half-life of bradykinin and potentially worsen HAE disease severity. Decreased levels of APP have been detected in patients on ACE inhibitors who develop angioedema[44]. Interestingly, long-term prophylaxis treatment of HAE with danazol results in increased APP activity,[45] suggesting that long-term prophylaxis therapy with danazol may improve symptoms, in part, by increasing bradykinin catabolism.

Bradykinin is a pluripotent nanopeptide that mediates a variety of physiologic and pathologic effects (reviewed in [46]). Binding of bradykinin to the bradykinin B2 receptor on vascular endothelial cells results in a marked increase in microvascular permeability. Lung et al[47] reported that HAE clinical severity is influenced by a polymorphism in the noncoding first exon of the bradykinin B2 receptor that affects bradykinin B2 receptor expression. Although a subsequent study failed to observe this pattern in a different cohort,[48] other studies have confirmed the role of this polymorphism in modulating bradykinin actions[49,50]. Additionally, a recent report suggested that the permeability enhancement in HAE attacks may be transduced by a combination of bradykinin B2 receptors and induced bradykinin B1 receptors[51].

The mechanism by which bradykinin enhances vascular permeability is thought to primarily involve its effects on the phosphorylation of vascular endothelial cell cadherin (VE-cadherin). VE-cadherin is the key protein involved in the formation of endothelial tight junctions, which regulate water movement across the endothelial layer. Bradykinin activates phospholipase-C, leading to increases in intracellular calcium and diacylglycerol (DAG), and activating protein kinase C. Protein kinase C phosphorylates beta-catenin and leads to the internalization and destruction of the VE-cadherin; it is also involved in the generation of the vasodilator nitric oxide[52]. Activated protein kinase C also phosphorylates myosin light chain kinase, promoting actin cytoskeleton contraction. Therefore, bradykinin causes the glue between the cells to disappear and causing the cells to centripetally contract. The net effect of this is to increase the gap between vascular endothelial cells, allowing water to move from the vascular space into the tissue. Clinically, this is angioedema.

In conclusion, HAE results from mutations in the SERPING1 gene, leading to production of a mutant C1 inhibitor protein that is either not secreted (type I HAE) or dysfunctional (type II HAE). The lack of sufficient functional C1 inhibitor in plasma to adequately inhibit the contact system proteases plasma kallikrein and coagulation factor XIIa results in increased generation of bradykinin, which acts on bradykinin B2 receptors to enhance vascular permeability.
